# A Case of Traumatic Tetanus

**Published:** 1850-04

**Authors:** Samuel S. Emison

**Affiliations:** Scott county, Ky.


Art. IV.—*A Case of Traumatic Tetanus.—Recovery.* By Samuel S. Emison, of Scott county, Ky.

Wm. P., æt. twenty years, very healty and athletic, was accidentally shot on the 25th December, 1849; the tow, with which the pistol was nearly filled, entered the left leg a little above and behind the external malleolus. Several large wads of tow were extracted soon after the receipt of the injury, and his medical attendant, believing that all foreign matter had been removed, directed him to poultice the wound, keep the limb quiet, and take a cathartic occasionally. In a few days the wound commenced suppurating, and another large piece of tow was removed. Soon after this, the wound healing rapidly, he disregarded the instructions which had been given. On the 13th January he rode several miles on horseback, the weather being cool and damp, and at night he felt considerable pain in the wound, with cramps of the muscles of the leg. On the 14th, these increasing, he was bled ℥xvi. without relief. His suffering gradually increased, and on the morning of the 15th, when I first saw him, there was constant rigidity of the muscles of the inferior extremities and abdomen. In addition, there were paroxysms of spasmodic contractions of the diaphragm, muscles of the back, and superior extremities, recurring every twenty minutes and producing well marked opisthotonos. These lasted but a few seconds each time. The skin was bathed in perspiration, bowels costive, pulse and respiration, during the intervals of the paroxysms, respectively ninety and twenty; urine scanty, high colored and voided with difficulty, tongue slightly coated, otherwise healthy; the patient had slept none the preceding night. The wound had nearly healed, but was dry, with everted edges; ancle somewhat swelled. At 10 o'clock, A. M., a powder, composed of calomel gr. xxv, ipecac gr. ij, was given, and a poultice of hops applied to the wound. At 12 o'clock, A. M., castor oil ℥jss, was given. An hour and a half after, a copious, feculent stool was procured, and soon after another. After the first, sulph. morphia, in doses of grs. ss., was given every half hour, for two hours, and afterwards laudanum gtt. lx every hour. After the third portion of laudanum, a disposition for sleep was manifested during the intervals of the alternate spasms, and the anodyne was suspended. No impression was made on the convulsions, which increased in severity, until 12 o'clock, P. M., when there appeared to be an abatement. The patient was directed to take the laudanum sufficiently often to keep him quite sleepy. Eleven o'clock, A. M., 15th, spasms again becoming severe; the treatment of the previous day was continued, except that the anodyne was given more freely, and a blister plaster substituted for the poultice. The cathartic procured but one small stool. The spasms were as frequent and more violent and painful than they had been during the previous evening. About midnight they again somewhat abated, but the exacerbation was renewed about 10 o'clock, A. M., 16th. The sufferer was now much exhausted; pulse one hundred and five and weak; skin perspiring freely; no appetite. The blister was dressed with basilicon ointment, and the laudanum given, *pro re nata.* The condition of the patient was now most distressing; the spasms were excited by every sudden noise, and every movement of his body. The remedies which had been employed, seeming to give little relief, if any, at 6 o'clock, P. M., 16th; sulph. quinine ʒss. was given, and the anodyne treatment was continued. There was a remission at midnight which continuing at 12 A. M., 17th, rhei. pulv. grs. xl, was given, and three hours after, castor oil ℥ij; acted freely. The anodyne was given p. r. n., and at 6 o'clock, P. M., sulph. quinine ʒi., was given. Ten o'clock, A. M. 18th, skin not too moist, pulse eighty, with more volume, periodic spasms very much less severe and not recurring oftener than once in two or three hours; some appetite; sleeps better. Laudanum given less frequently, and at 6 P. M. quinine ʒss. Ten A. M., 19th, spasms return at distant intervals, and very slight, except in the muscles of the abdomen; that region is still almost as hard as a board. The convulsive movements, which now seldom take place, except when the patient attempts to turn in bed, very much resemble those produced by a light shock from electricity. The treatment of the past few days, detailed above, continued four or five days longer, during which time the spasms entirely disappeared. The wound was made to suppurate with some difficulty, but finally healed and the man is now well.

I have given a faithful and by no means an exaggerated account of what I considered a case of traumatic tetanus, arrested mainly by the use of quinine. Others may draw different conclusions. It may be said that the large doses of opium and mercury were the better remedies; or, that the vis medicatrix naturæ achieved a victory over both disease and empiricism combined. I confess that it was with little hope of benefit that I gave the quinine, nor do I claim originality for the use of the article in such cases. The practice originated with one of the oldest and most eminent surgeons of our country. At all events it would not be amiss to give quinine a fair trial, in a disease which has been so long one of the opprobria of the profession.

Scott county, Ky., Feb., 1850.

